# Privacy and the acceptance of centralized digital currencies in the U.S., India and Germany

**DOI:** 10.1038/s41598-023-35905-y

**Published:** 2023-05-30

**Authors:** Guido Mehlkop, Robert Neumann, Hagen von Hermanni

**Affiliations:** 1 Chair in Methods of Empirical Social Research, Faculty of Economics, Law and Social Sciences, University of Erfurt, PO Box 90 02 21, 99105 Erfurt, Germany; 2 Chair in Methods of Empirical Social Research, Technische Universität Dresden, 01062 Dresden, Germany

**Keywords:** Psychology, Human behaviour

## Abstract

National governments around the world increasingly acknowledge the possibility of introducing new digital forms of money and implementing policies that trigger their adoption. Knowledge about the acceptance of such measures, however, is rather limited. Next to the regulatory uncertainty about the impact of a Central Bank Digital Currency (CBDC) on competition, on financial stability and questions on the integrity and technical implementations of a CBDC, recent announcements of the joint venture of e.g. the European Central Bank with a large and globally operating private company emphatically raise questions about data privacy. Therefore, we report results of a survey experiment conducted in the United States of America, Germany and India to investigate the acceptance of an app-based monthly digital payment similar to a Universal Basic Income and investigate its adoption across income levels. Controlling for privacy features and short-term vs. long-term incentives to adopt the digital payment app, we find strong reservations with regard to the involvement of multinational tech companies in establishing new digital mediums of exchange, while also finding contextual differences in acceptance levels between the studied populations.

## Introduction

Global trends such as the digitalization of economic transactions and the increasing importance of data processing manifest themselves by a decrease of cash payments and are accompanied by ideas such as the introduction of new forms of electronic money. The push for new digital currencies is driven by private companies, national governments and central banks alike. While decentralized solutions like Bitcoin, innovations in the area of decentralized finance (DEFI) and private efforts to establish asset-backed stablecoins (e.g. USD Coin or USD Tether) have already put pressure on traditional financial entities to accelerate their investments in the area of financial technology innovations (Fintech), governments and central banks play a much more important role in rethinking potential new forms of money and the future of monetary supply. In particular, the idea of a Central Bank Digital Currency (CBDC) has gained traction among several central banks across the world, with 56 entities conducting research on the subject and two CBDCs already having been launched in the last year^1,2^.

The potential for private consumers to directly open up central bank accounts—independently of commercial banks—might sound intriguing with regard to providing financial stability and enable financial inclusion. In the aftermath of the recent COVID-19 pandemic, governments around the world have to shoulder the burden of increasing budget deficits^3,4^, high levels and unequal distribution of sovereign debts, rising pressures from high inflation and concerns about potential credit defaults^5,6^. In times of crisis, central banks serve the role of last-resort lenders, providing liquidity and third party trust for the participants of credit and financial markets. Still, central bank mandates usually remain restricted to maintain price stability and do not involve direct interference with consumers.

Amongst others, the European Central Bank (ECB) recently unveiled a plan to introduce a pilot for a CBDC that provides a potential blueprint for how central banks worldwide might enable citizens’ access to a new digital currency through secondary payment channels. In fact, Christine Lagarde presented the case for a CBDC in a speech in November 2018 by emphasizing its positive effects on “…financial inclusion, security and consumer protection; and to provide what the private sector cannot: privacy in payments”^7^. Surprisingly, one key difference in the announced pilot project of the ECB is the involvement of the private multinational company Amazon to conduct the pilot study with regard to the technical feasibility of a digital euro. Next to the regulatory uncertainty about the impact of a CBDC on competition and financial stability^8^ and questions on the integrity and technical implementations of a CBDC, the announcement of the joint venture with a large and globally operating private company emphatically raises questions about data privacy.

Current research on new forms of digital currencies in general and CBDCs in particular are mainly focused on welfare and policy implications, in particular how the introduction of a CBDC would affect competition in the banking sector and on the credit and lending markets^8,9^. Next to the largest economy in the world and the largest economy in Europe, we choose India as an additional population to account for the rising criticism on conducting experiments only on populations from so-called WEIRD countries (Western, Educated, Industrialized, Rich, and Democratic^10^). Crucially, only some scholars have studied the interplay of privacy-preserving techniques along with the introduction of digital currencies. For instance, economists have argued that under an optimally designed and privacy-preserving solution, a digital currency would increase social welfare by allowing optimal inferences about consumer preferences on the basis of observed consumer choices^11,12^. Based on the assumption that a privacy-preserving architecture can actually be established, they underscore the key requirement for the success of electronic cash: The individual burden of preserving privacy has to be lower than the perceived benefit of using new electronic forms of cash^11^.

Against this background, there remains a research gap regarding under which conditions citizens would actually accept and use new forms of digital currencies instead of cash. These questions tap into the area of privacy value research, where studies have assessed the monetary values individuals assign to the preservation of privacy of different data types by means of conjoint analysis or discrete choice experiments^13,14^. For instance, a recent comparative study across six countries investigated different data types and found that respondents value the privacy of bank balances and fingerprints the highest, while females and older respondents assign higher monetary valuations for the preservation of privacy^15^. These studies often apply the concepts of willingness-to-pay (WTP) and willingness-to-accept (WTA) for a valuation of privacy, hence they assign monetary values to the value of privacy^16^. Instead, we are interested in behavioral measures to investigate the conditions under which actors would use new digital currencies and whether they are more concerned with possible privacy violations, despite being incentivized to give up their privacy rights for long or short term benefits^17^.

Our study addresses these questions by means of an experimental factorial design that we embedded in a cross-country online survey conducted in the summer of 2022 in the United States (U.S.), India and Germany. We use data from 3532 interviews and 10,592 choice situations to investigate the conditions under which citizens decide in favor of a digital currency instead of cash. In particular, we model the introduction and subjective benefits of a digital currency in two distinct ways. First, we address the open question of how an optimal design would look to convince consumers to adopt a digital currency in practice by making its usage conditional to a windfall gain: a monthly payment of a universal basic income (UBI). Second, we model the conditions under which the UBI can be spent digitally or paid out in cash by varying the monetary incentives to use the UBI digitally: We tie spending of the UBI to the adoption of an app that is used for payment where consumers either (a) do not receive any special benefits (except of convenience), (b) they can receive a rebate on their VAT payment, or (c) consumers receive an automatic payment to a private mutual pension fund. The design enables us to investigate whether consumers accept digital currencies given short-term (VAT rebate) or long-term benefits (consumption based retirement saving). Together, both approaches come with the advantage that the utility derived from the adoption of the digital currency remains independent of the adoption of others, therefore we can model the perceived benefits from individual adoption independent of network effects. Third, we vary the proximity as to how feedback on consumptions patterns is communicated to private consumers. In addition to no feedback about such data processing, the app provider within the experiment either processed the consumption data remotely or provided feedback via a monthly overview about all things purchased via the app. The closer proximity of the information via a postal letter can be considered more invasive with regard to privacy rights since the home address is involved more prominently in the app adoption, touching upon privacy concerns related to locality and place^18^.

Additionally, we scrutinize the role of the app provider and its influence on the acceptance of a digital currency as well as the importance of descriptive norms on the adoption of the UBI app. The former covers the question as to whether consumers perceive the introduction of the UBI app by a private, governmental or supra-governmental differently by presenting the app provider as either the nation’s ministry of finance, the local central bank or a multinational internet corporation. The latter inspects whether the social expectations about the behavior of other consumers affect the decision to use the UBI app for payments in contrast to choosing the privacy-preserving option by cashing out the monthly UBI payment. The power of social norms on consumer choices has already been demonstrated in other research contexts^19,20^ and we consider it an important antecedent for whether consumers will pick up new digital payment solutions.

## Results

**Figure 1:**
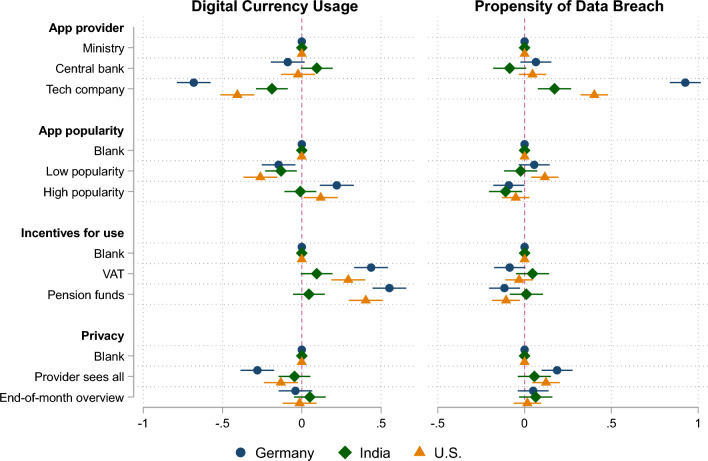
Regression coefficients plotted for the main experimental variables (with 95% confidence intervals).

Our results displayed in Fig. 1 reveal important insights: We find that the introduction of a digital currency via a UBI app by a multinational internet company leads consumers to opt much more strongly in favor of privacy-preserving cash, in contrast to if the app provider is a branch the national government or the respective central bank (the FED, vs. Central Bank of India vs. ECB). We also find a strong impact of descriptive norms for the U.S. and Germany: If app adoption is framed as popular and used by many others, respondents are more likely to adopt the digital currency through the app than to opt for cash.

Turning to the problem of incentivizing the adoption of digital currencies via the app, we find that long-term incentives in the form of funding a retirement fund represent a slightly stronger incentive to use the app than a short-term VAT rebate. Interestingly, both incentives show no effect on app usage or the cash option in the Indian sample.

We also model different privacy features implemented in the UBI app and asked respondents how likely it is that their private consumer data is ineligibly shared with third parties. On the one hand, German and American respondents consider the data trail about the purchased items with the app provider as problematic in the sense that they more frequently opt for cash while also considering data breaches being more likely under these circumstances. Information feedback about the monthly purchases sent to the postal address at home does not play a role—neither for app adoption nor for the perceived likelihood of privacy violations. Surprisingly, rather invasive feedback mechanisms involving a home address does not seem to bother respondents at all.

One general finding is that respondents from India are much more likely to adapt the app and use the new digital currency, while not being responsive to the short term or long term incentives. First, a possible explanation for these results can be traced back to the push by the Indian government to get rid of traditional cash and facilitate electronic payments. In fact, in 2016 they removed 86% percent of all value of notes in circulation^21^. Second, the Reserve Bank of India even introduced a mobile payment application similar to the idea within our factorial survey design denoted Unified Payments Interface (UPI) which enables instant money transfers between bank accounts using mobile devices as well as between vendors and customers^22^. Hence, one might assume that there is a larger degree of familiarity among Indian respondents with regard to such an app and they are actually less concerned of using the service.

Second, cultural explanations could be brought forward to explain why neither long nor short term incentive appear to have an effect on digital currency usage. Due to the religious belief system established in India (mostly Hinduism along with cast system), inequality and unequal levels of consumption may be perceived as more legitimate than in countries from the “Western” hemisphere^23^. Hence, long term accumulation of wealth through contributions to a pension funds might not represent an incentive worth considering. In contrast, concepts like karma, self-control and detachment provide the prerequisites to act rather independent of short term incentives or consequences. These contradicting mechanisms may play a role in the null effect we find in our experiment.

**Figure 2:**
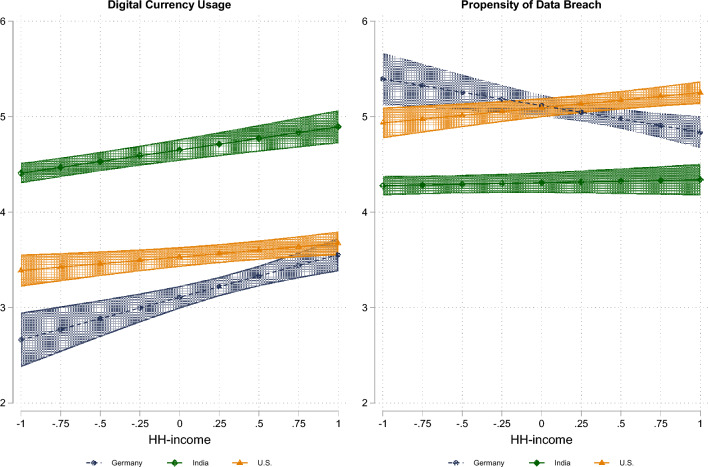
Adoption of an app-based digital currency instead of opting for a cash payout, plotted conditional to the purchasing power adjusted net equalized household income (z-standardized). We find increasing digital currency usage given higher household incomes in Germany and India, but no substantial difference in app adoption across income groups in the U.S. Note the higher acceptance levels in in India, marked by less scrutiny about potential privacy violations.

In order to prevent personal characteristics from confounding our results, we additionally tested hierarchical linear regression models with country fixed effects and included interaction effects to control for the varying impact of income across the contexts. Monthly windfall gains like the UBI are perceived as especially beneficial for part-time and/or non-permanently employed persons that can be assumed to belong to low income households^24^. But it remains an open question whether low-status individuals would therefore be more in favor of using the digital currency via the app. Figure 2 illustrates that there is substantial variation across income levels and contexts. In Germany and India, we find that rising net adjusted household income is associated with higher acceptance of the digital currency via the app. Overall, German respondents show the lowest overall level of digital currency acceptance, replicating the results of other studies^15^. In contrast, respondents from the U.S. do not differ in their preference for cash or app use, conditional to their income. This finding also holds true for their perception of the likelihood of privacy violations, while the pattern among Indian and German respondents is more complex. First, Indian respondents are generally less concerned about privacy violations than their American and German counterparts. Second, Indian households with high net equivalent incomes consider data breaches as being less likely than households with below-average incomes, while German respondents reveal the opposite pattern. Again, the results for India could be explained by a lack of negative experiences with the new payment solutions that were introduced in India in 2016^25^.

Additional robustness analyses reveal that our findings are not mediated through variables covering institutional trust (see the Online Supplementary Information). Both the country-specific analysis and the multilevel model with country fixed effects show the robustness of the coefficients of our experimental variables, even after controlling for the standard trust based measures (measured with item-specific 11-point scales) with regard to the ministries, central banks and federal governments in the respective countries. In particular, the higher propensity for respondents in India to opt for the app usage across all income groups reveals a substantial difference in the possible merits of digital currencies.

## Discussion

National governments and supra-national institutions that are considering the introduction of a parallel digital currency alongside standard fiat money still have close to no information about the level of acceptance of such a medium of exchange as well as no first best solution regarding how to get citizens involved in the project. First, we were able to show that the introduction of a digital currency by means of an app-based UBI can find support within the population, despite obvious concerns about data privacy and violations by third parties. Especially in India, acceptance of a digital currency distributed via a UBI app is substantially higher than in Germany or the U.S. Another key finding from our cross-country survey experiment is that the introduction of digital currency with help of Big Tech platform companies such as Amazon, Google or Meta will be perceived as a less trustworthy currency solution. Similar results have been generated for the U.S. and the trustworthiness of Big Tech with regard to the storage of private data^26^. Together, this implies that joint ventures with large and globally operating tech companies may be ill advised to trigger acceptance or achieve adoption of new forms of digital money. In contrast, both short-term and long-term incentives shape an opportunity structure that facilitates user adoption, even to a larger extent than the behavioral intentions of other users. Further research on the acceptance of digital currencies should follow upon the results presented here by either investigating the popularity of different (private) app providers other than Big Tech firms (e.g. national brands), studying the acceptance of alternative decentralized versions of digital currencies (e.g. DEFI protocols) or by varying the monthly amount of an UBI. Policy makers will benefit from such experimental investigations, because they provide insights about the consequences of e.g. behaviorally informed designs that represent non-invasive forms of regulations through choice architecture and have been shown to be quite effective across several domains^27^.

## Methods

The research data for this article was collected by the private survey data provider Bilendi & respondi. All procedures performed in this study adhere to the ethical standards of the 1964 Helsinki Declaration and its later amendments or comparable ethical standards. The data was collected following institutional guidelines of ESOMAR21 as well as the international quality norm ISO 20252:2019. All participants provided informed consent to take part in this study. Study participation did not affect the physical or psychological integrity, the right for privacy, or other personal rights or interests of the participants. Since the data was collected by a certified survey data provider, no deception was used and no personalized information was collected, the study did not meet the formal characteristics that require a formal approval from Ethics Commission at Technische Universität Dresden (§1, Abs., No. 1.-3. Statute of the Ethics Committee, TU Dresden) and therefore approved by the DPO of TU Dresden.

The interviewees for our self-administrated online survey with integrated factorial-survey experiments were recruited by Bilendi & respondi from three access panels in India, USA and Germany. The members of these access panels were sampled using both the online as well as the telephone mode of recruitment. Respondents were invited to the self-administered web-survey using a crossed quota design with respect to age and gender to ensure representativeness within each country. Together, we use data from 1194 interviews from the United States, 1172 from India and 1166 from Germany. Our experimental design was implemented in all three country surveys, in which we changed the description of the national services and institutions in accordance to the national idiosyncrasies. The fully confounded factorial vignette design^28^ comprised four dimensions (app provider, popularity, privacy and incentives for usage) with three levels each, resulting in a vignette universe of 81 possible combinations; 27 sets with three vignettes each were arranged orthogonally and were randomly assigned to respondents within each country sample. The main dependent variable was asked after each vignette and stated “Would you use the described app to pay or get the money in cash instead?” Acceptance of the digital payment method was measured on a 7-point item-specific scale (1 “always pay with app” to 7 “always pay out cash”). For the analysis we recoded this scale so that larger values reflect higher acceptance of digital currencies via app usage. Afterwards, a second question asked about the likelihood that the personal information will be shared with third parties without permission (1 “not at all likely” to 7 “highly likely”). Full wording of a vignette is shown in the Online SI.

Income figures were equalized using purchasing power parity conversion rates from the year 2021, adjusted for household size^29^; these entered the models in standardized form after taking the natural logarithm. Institutional trust was measured in accordance to standard approaches and was assessed on an 11-point Likert scale (0 “No trust at all” to 10 “Complete trust”). In general, all models control for age, squared age and gender.

## Supplementary Information

Supplementary Information.

## Data Availability

The experimental data and do-files will be made available upon request. Contact for data request: Robert Neumann (robert.neumann@tu-dresden.de).

## References

[1] Auer R (2022). Central bank digital currencies: Motives, economic implications, and the research frontier. Annu. Rev. Econ..

[2] Morgan J (2022). Systemic stablecoin and the defensive case for Central Bank Digital Currency: A critique of the Bank of England’s framing. Res. Int. Bus. Financ..

[3] Makin AJ, Layton A (2021). The global fiscal response to COVID-19: Risks and repercussions. Econ. Anal. Policy.

[4] Tevdovski D, Jolakoski P, Stojkoski V (2022). Determinants of budget deficits: The effects of the COVID-19 crisis. Econ. Ann..

[5] Dalio R (2022). Principles for Navigating Big Debt Crises.

[6] Jahromi, A. A., Mihai, M. M. & Yang, T. Inflation and the US economy in 2022. *J. Financ. Serv. Prof.* **77** (2023).

[7] Lagarde, C. Winds of change: The case for new digital currency. https://www.imf.org/en/News/Articles/2018/11/13/sp111418-winds-of-change-the-case-for-new-digital-currency

[8] Fernández-Villaverde J, Sanches D, Schilling L, Uhlig H (2021). Central bank digital currency: Central banking for all?. Rev. Econ. Dyn..

[9] Williamson S (2022). Central bank digital currency: Welfare and policy implications. J. Polit. Econ..

[10] Henrich J (2020). The WEIRDest People in the World: How the West Became Psychologically Peculiar and Particularly Prosperous.

[11] Garratt RJ, Van Oordt MRC (2021). Privacy as a public good: A case for electronic cash. J. Polit. Econ..

[12] Ren, D., Guo, H. & Jiang, T. Managed anonymity of CBDC, social welfare and taxation: A new monetarist perspective. *Appl. Econ.* 1–22 (2022).

[13] Savage SJ, Waldman DM (2015). Privacy tradeoffs in smartphone applications. Econ. Lett..

[14] Spiekermann S, Korunovska J (2017). Towards a value theory for personal data. J. Inf. Technol..

[15] Prince JT, Wallsten S (2022). How much is privacy worth around the world and across platforms?. J. Econ. Manag. Strateg..

[16] Chapman, J., Dean, M., Ortoleva, P., Snowberg, E. & Camerer, C. Willingness to pay and willingness to accept are probably less correlated than you think (2017).

[17] Acquisti A, Brandimarte L, Loewenstein G (2015). Privacy and human behavior in the age of information. Science.

[18] Martin K, Nissenbaum H (2020). What is it about location?. Berkeley Tech. LJ.

[19] Allcott H (2011). Social norms and energy conservation. J. Public Econ..

[20] Przepiorka W, Horne C (2020). How can consumer trust in energy utilities be increased? The effectiveness of prosocial, proenvironmental, and service-oriented investments as signals of trustworthiness. Organ. Environ..

[21] Ghosh J, Chandrasekhar CP, Patnaik P (2017). Demonetisation Decoded: A Critique of India’s Currency Experiment.

[22] Ramesh, G., Jangid, A., Sivamalai, L. & Rebelly, A. B. NPCI: Chartering a payment freeway. *IIM Bangalore Res. Pap.* (2020).

[23] Pellissery S, Pampackal AJ, Bopaiah P (2015). Caste and distributive justice: Can social policy address durable inequalities?. Soc. Policy Adm..

[24] Kangas O, Jauhiainen S, Simanainen M, Ylikanno M (2021). Experimenting with Unconditional Basic Income.

[25] D’souza, R. Cashless India: Getting incentives right. *ORF Occas. Pap. Mumbai Obs. Res. Found.* (2018).

[26] Armantier, O., Doerr, S., Frost, J.,* Fuster, A. & Shue, K. Whom do consumers trust with their data? US survey evidence* (2021).

[27] Mertens S, Herberz M, Hahnel UJJ, Brosch T (2022). The effectiveness of nudging: A meta-analysis of choice architecture interventions across behavioral domains. Proc. Natl. Acad. Sci..

[28] Auspurg K, Hinz T (2015). Factorial Survey Experiments.

[29] OECD. What are equivalence scales? *OECD Proj. Income Distrib. Poverty* (2011).

