# Supplementary material for: Privacy and the acceptance of centralized digital currencies in the U.S., India and Germany
Source: Sci Rep. 2023 May 30;13:8772. doi: 10.1038/s41598-023-35905-y (PMC10228898; doi:10.1038/s41598-023-35905-y)
Supplement: Supplementary file 1 — Supplementary Information. [file 41598_2023_35905_MOESM1_ESM.docx]

Supporting Information for

**Privacy and the Acceptance of Centralized Digital Currencies in the U.S., India and Germany**

Guido Mehlkop, Robert Neumann, Hagen von Hermanni

Corresponding author: Guido Mehlkop

Email: guido.mehlkop@uni-erfurt.de

Sample Description

The data used in this paper consist of three cross-sectional data sets that were collected in the months of August and September 2022 in Germany, India and the United States as an internet-based web survey. The sample recruitment and data collection along with the quality assurance process were conducted by Bilendi & respondi. The sample recruitment was based on a crossed quota design between gender and seven age groups to ensure country-specific representativeness. The eligible range for respondents was from 18 to 70 years of age. The composition of the final samples is shown in Table S1.

**Table S1: Country-specific Descriptive Statistics and Sample Compositions**

**Germany**

| **Variable** | **Obs.** | **Mean** | **Std. Dev.** | **Min** | **Max** |
| --- | --- | --- | --- | --- | --- |
| Age | 1,166 | 45.648 | 14.508 | 18 | 70 |
| Log (household income) | 1,165 | 7.797 | .626 | 5.262 | 9.51 |
| Socioeconomic Status | 1,164 | 5.503 | 2.132 | 1 | 11 |
| Trust in Multinational Corp. | 1,126 | 4.407 | 2.562 | 1 | 11 |
| Trust in Central Bank (ECB) | 1,077 | 5.509 | 2.848 | 1 | 11 |
| Trust in Ministry of Finance | 1,086 | 5.452 | 2.834 | 1 | 11 |

|  | Abs. Freq. | Relative Frequency (in Percent) | Cumulative |  |
| --- | --- | --- | --- | --- |
| Sex |  |  |  | |
| male | 578 | 49.57 | 49.57 | |
| female | 588 | 50.43 | 100.00 | |
| Total | 1,166 | 100.00 |  | |

**India**

| **Variable** | **Obs.** | **Mean** | **Std. Dev.** | **Min** | **Max** |
| --- | --- | --- | --- | --- | --- |
| Age | 1,173 | 43.942 | 14.277 | 18 | 70 |
| Log (household income) | 1,164 | 6.62 | 1.465 | -.51 | 10.204 |
| Socioeconomic Status | 1,171 | 6.886 | 2.251 | 1 | 11 |
| Trust in Multinational Corp. | 1,150 | 7.883 | 2.39 | 1 | 11 |
| Trust in Central Bank (RBI) | 1,151 | 8.849 | 2.236 | 1 | 11 |
| Trust in Ministry of Finance | 1,127 | 7.711 | 2.741 | 1 | 11 |

|  | Abs. Freq. | Relative Frequency (in Percent) | Cumulative |  |
| --- | --- | --- | --- | --- |
| Sex |  |  |  | |
| male | 617 | 52.60 | 52.60 | |
| female | 556 | 47.40 | 100.00 | |
| Total | 1,173 | 100.00 |  | |

**U.S.**

| **Variable** | **Obs.** | **Mean** | **Std. Dev.** | **Min** | **Max** |
| --- | --- | --- | --- | --- | --- |
| Age | 1,194 | 45.539 | 14.64 | 18 | 69 |
| Log (household income) | 1,192 | 7.783 | 1.219 | 2.323 | 10.389 |
| Socioeconomic Status | 1,191 | 5.715 | 2.362 | 1 | 11 |
| Trust in Multinational Corp. | 1,152 | 5.266 | 2.936 | 1 | 11 |
| Trust in Central Bank (FED) | 1,086 | 6.238 | 2.989 | 1 | 11 |
| Trust in Ministry of Finance | 1,111 | 6.28 | 2.947 | 1 | 11 |

|  | Abs. Freq. | Relative Frequency (in Percent) | Cumulative |  |
| --- | --- | --- | --- | --- |
| Sex |  |  |  | |
| male | 579 | 48.49 | 48.49 | |
| female | 615 | 51.51 | 100.00 | |
| Total | 1,194 | 100.00 |  | |

**Details of the experiment**

The vignette design was conceptualized as a fully confounded factorial design that allows for both within- and between-respondent analyses. First, we specified that each respondent had to read and answer three vignettes that described the introduction of the UBI via an app provided by different institutional entities with varying popularity and different privacy features. In detail, the vignettes were varied across four dimensions with three levels for each dimension. Participants were randomly assigned to one of the 27 vignette sets that were each comprised of three vignettes per respondent.

An introduction to the decision problem was presented as follows (for the U.S. sample): “An unconditional basic income of 1,000.00 U.S.-Dollars per month is introduced in the United States of America, which is open to all citizens in addition to their wages, salaries, pensions and all other social benefits. In order to receive this basic income of 1,000.00 U.S.-Dollars, you need a smartphone and must register with a specific app. With the app, you either can pay everywhere or make a cash withdrawal at ATMs.”

“To receive the unconditional basic income of 1,000.00 USD per month, you must register with a specific app. The provider of the app is the United States Department of the Treasury. If you pay with the app, then you receive a credit for your retirement savings (deposit of 20% of the VAT amount into a retirement fund). A pilot study has shown that almost no one wants to use the app for payment. At the end of each month, you will receive a detailed overview (date, place, time) by mail of what exactly you paid for with the app.”

**Table S2: Vignette Dimensions and Country-specific Vignette-level Wording**

|  | **App Provider** | **App Popularity** | **Incentive** | **Privacy** |
| --- | --- | --- | --- | --- |
| **Germany*** | Bundesfinanz-ministerium | No information (blank). | No incentive (blank). | No feedback (blank). |
|  | Internet-Konsortium, bestehend aus Meta (Facebook), Alphabet (Google) und Apple | Eine Pilotstudie hat gezeigt, dass nur wenige Menschen mit dieser App bezahlen wollen. | Beim Bezahlen mit der App erhalten Sie auf alle Produkte einen Rabatt von 20% der Mehrwertsteuer | Alle Ihre Einkäufe sind für den Betreiber der App lückenlos einsehbar. |
|  | Die Europäische Zentralbank (EZB) | Eine Pilotstudie hat gezeigt, dass viele Menschen mit dieser App auch bezahlen wollen. | Wenn Sie mit der App bezahlen, dann erhalten Sie eine Gutschrift für ihre Altersvorsorge (Einzahlung von 20% der Mehrwertsteuer in einen Rentenfonds). | Sie erhalten am Ende jeden Monats eine detaillierte Übersicht (Datum, Ort, Uhrzeit) per Post, was genau sie mit der App bezahlt haben. |
| **United States of America** | The United States Department of the Treasury | No information (blank). | No incentive (blank). | No feedback (blank). |
|  | An internet consortium consisting of Meta (Facebook), Alphabet (Google) and Apple | A pilot study has shown that almost no one wants to use the app for payment. | When paying with the app, you will receive a 20% VAT discount on all products. | All your purchases are fully visible to the app operator. |
|  | The Federal Reserve (also known as the Fed) | A pilot study has shown that many people want to use the app for payment. | If you pay with the app, then you get a credit for your retirement savings (deposit of 20% of the VAT amount into a retirement fund). | At the end of each month, you will receive a detailed overview (date, place, time) by mail of what exactly you paid for with the app. |

| Table S2 continued | |  | |  | |  | |
| --- | --- | --- | --- | --- | --- | --- | --- |
|  | **App Provider** | | **App Popularity** | | **Incentive** | | **Privacy** |
| India** | the Ministry of Finance of India | | No information (blank). | | No incentive (blank). | | No feedback (blank). |
|  | An internet consortium consisting of Meta (Facebook), Alphabet (Google) and Apple | | A pilot study has shown that almost no one wants to use the app for payment. | | When paying with the app, you will receive a 20% VAT discount on all products. | | All your purchases are fully visible to the app operator. |
|  | The Reserve Bank of India (RBI) | | A pilot study has shown that many people want to use the app for payment. | | If you pay with the app, then you get a credit for your retirement savings (deposit of 20% of the VAT amount into a retirement fund). | | At the end of each month, you will receive a detailed overview (date, place, time) by mail of what exactly you paid for with the app. |

Note:

* The amount for the UBI was 1,000.00 Euro per month for German respondents.

**The amount for the UBI was 26,500 INR per month for Indian respondents.

**Additional explanatory variables**

Household income was first measured by an open-ended question about the total monthly income of the entire household, including all transfers, net after taxes. If no information was given, a closed form comprised of 16 income groups was presented. We took the midpoint of each income category as the reference in case no exact income figures were reported. We then performed the following transformations: First, we equalized the income dollars by converting all figures into U.S. dollars using purchasing power parity conversion rates for the year 2021 provided by the OECD ^1^. Second, we used the number of household members to compute net equivalent household income figures using the square root formula ^2^. Third, after taking the natural logarithm, we standardized the income figure. A small number of households from India reported very low to no income, hence the transformations yields negative values for the log income figures. We performed all model estimations on a reduced sample without these low to no income households and results do not change at all. Ultimately, we decided against dropping these cases.

Subjective socioeconomic status was measured via the MacArthur Scale of Subjective Social Status, asking respondents where they would place themselves on a scale ranging from 0 (“Bottom of our society”) to 10 (“Top of our society”), anchored by the visual presentation of a vertical ladder.

The measure of institutional trust was based on nation-specific formulation. Hence, item-based measures of institutional trust were varied across the respective country questionnaires in accordance to the nomenclature of the national institutions. For the U.S., we assessed institutional trust with regard to the *United States Department of the Treasury* and the *Federal Reserve Bank*. For India, we asked for the trust in the *Ministry of Finance of India* and the *Reserve Bank of India*. For Germany, we assessed the trust in the *Bundesfinanzministerium* and the *European Central Bank*, respectively. The trust in *multinational internet corporations (e.g. Alphabet/Google* and *Meta/Facebook)* was not varied between the countries. The question on trust in institutions followed standardized design templates and asked: “How much do you personally trust the following organizations and institutions?” with possible answers ranging from 0 = “*No trust at all*” to 10 = “*Complete trust*”.

Table S3: Estimation Results for the Country-specific Analyses for Germany

|  | Digital Currency Usage | | | Propensity of Data Breach | | |
| --- | --- | --- | --- | --- | --- | --- |
|  | M1 | M2 | M3 | M4 | M5 | M6 |
| Central Bank | -0.0888 | -0.0837 | -0.0385 | 0.0653 | 0.0602 | -0.0212 |
|  | (-1.63) | (-1.54) | (-0.65) | (1.44) | (1.33) | (-0.43) |
| Tech Company | -0.682^***^ | -0.678^***^ | -0.659^***^ | 0.926^***^ | 0.927^***^ | 0.880^***^ |
|  | (-12.53) | (-12.48) | (-11.38) | (20.36) | (20.46) | (18.33) |
| Low Popularity | -0.147^**^ | -0.151^**^ | -0.169^**^ | 0.0557 | 0.0608 | 0.0429 |
|  | (-2.69) | (-2.79) | (-2.95) | (1.22) | (1.34) | (0.90) |
| High Popularity | 0.221^***^ | 0.220^***^ | 0.202^***^ | -0.0906^*^ | -0.0848 | -0.0808 |
|  | (4.05) | (4.05) | (3.52) | (-1.99) | (-1.87) | (-1.70) |
| VAT | 0.438^***^ | 0.434^***^ | 0.425^***^ | -0.0857 | -0.0866 | -0.0785 |
|  | (8.04) | (7.98) | (7.42) | (-1.88) | (-1.91) | (-1.65) |
| Pension Funds | 0.553^***^ | 0.554^***^ | 0.541^***^ | -0.116^*^ | -0.122^**^ | -0.107^*^ |
|  | (10.15) | (10.19) | (9.46) | (-2.55) | (-2.69) | (-2.25) |
| Provider Sees All | -0.280^***^ | -0.276^***^ | -0.287^***^ | 0.187^***^ | 0.188^***^ | 0.191^***^ |
|  | (-5.15) | (-5.08) | (-5.01) | (4.12) | (4.16) | (4.02) |
| End‑of‑Month Overview | -0.0403 | -0.0349 | -0.0275 | 0.0496 | 0.0446 | 0.0459 |
|  | (-0.74) | (-0.64) | (-0.48) | (1.09) | (0.98) | (0.97) |
| Male |  | 0.301^**^ | 0.321^**^ |  | -0.251^**^ | -0.263^**^ |
|  |  | (2.99) | (3.18) |  | (-2.78) | (-2.96) |
| Age (group z) |  | -0.485^***^ | -0.481^***^ |  | 0.159^***^ | 0.141^**^ |
|  |  | (-9.68) | (-9.48) |  | (3.55) | (3.17) |
| HH Income (group z) |  | 0.221^***^ | 0.201^***^ |  | -0.127^**^ | -0.108^*^ |
|  |  | (4.38) | (3.96) |  | (-2.82) | (-2.42) |
| Trust in Institutions (group z) |  |  | 0.203^***^ |  |  | -0.325^***^ |
|  |  |  | (4.05) |  |  | (-7.71) |
| Central Bank # Trust in Central Bank (group z) |  |  | -0.125^*^ |  |  | 0.181^***^ |
|  |  |  | (-1.97) |  |  | (3.40) |
| Tech Company # Trust in Tech Companies (group z) |  |  | -0.170^**^ |  |  | 0.308^***^ |
|  |  |  | (-2.93) |  |  | (6.36) |
| intercept | 3.244^***^ | 3.092^***^ | 3.087^***^ | 4.703^***^ | 4.830^***^ | 4.879^***^ |
|  | (39.33) | (32.57) | (31.42) | (67.32) | (58.58) | (57.92) |
|  |  |  |  |  |  |  |
| var(intercept) | 2.651^***^ | 2.350^***^ | 2.265^***^ | 2.001^***^ | 1.944^***^ | 1.813^***^ |
|  | (19.19) | (16.42) | (14.92) | (13.85) | (13.22) | (11.08) |
| var(Residual) | 1.724^***^ | 1.717^***^ | 1.757^***^ | 1.205^***^ | 1.193^***^ | 1.210^***^ |
|  | (18.59) | (18.43) | (18.31) | (6.36) | (6.04) | (6.16) |
| Observations | 3,496 | 3,493 | 3,284 | 3,497 | 3,494 | 3,285 |

*t* statistics in parentheses; ^*^ *p* < 0.05, ^**^ *p* < 0.01, ^***^ *p* < 0.001

Table S4: Estimation Results for the Country-specific Analyses for India

|  | Digital Currency Usage | | | Propensity of Data Breach | | |
| --- | --- | --- | --- | --- | --- | --- |
|  | M7 | M8 | M9 | M10 | M11 | M12 |
| Central Bank | 0.0944 | 0.0962 | 0.0560 | -0.0855 | -0.0801 | -0.0518 |
|  | (1.85) | (1.88) | (1.07) | (-1.76) | (-1.64) | (-1.03) |
| Tech Company | -0.188*** | -0.190*** | -0.256*** | 0.172*** | 0.180*** | 0.209*** |
|  | (-3.69) | (-3.72) | (-4.63) | (3.54) | (3.68) | (3.95) |
| Low Popularity | -0.131* | -0.140** | -0.140** | -0.0227 | -0.0228 | -0.0184 |
|  | (-2.57) | (-2.72) | (-2.70) | (-0.46) | (-0.47) | (-0.37) |
| High Popularity | -0.00934 | -0.0124 | -0.0218 | -0.110* | -0.115* | -0.101* |
|  | (-0.18) | (-0.24) | (-0.42) | (-2.25) | (-2.35) | (-2.05) |
| VAT | 0.0938 | 0.0888 | 0.106* | 0.0454 | 0.0495 | 0.0586 |
|  | (1.83) | (1.73) | (2.03) | (0.93) | (1.01) | (1.18) |
| Pension Funds | 0.0449 | 0.0418 | 0.0486 | 0.0104 | 0.0145 | 0.0139 |
|  | (0.88) | (0.82) | (0.94) | (0.21) | (0.30) | (0.28) |
| Provider Sees All | -0.0460 | -0.0492 | -0.0372 | 0.0565 | 0.0589 | 0.0637 |
|  | (-0.90) | (-0.96) | (-0.72) | (1.16) | (1.20) | (1.29) |
| End‑of‑Month Overview | 0.0504 | 0.0521 | 0.0507 | 0.0642 | 0.0586 | 0.0709 |
|  | (0.99) | (1.02) | (0.98) | (1.32) | (1.20) | (1.43) |
| Male |  | 0.245* | 0.236* |  | 0.153 | 0.152 |
|  |  | (2.38) | (2.30) |  | (1.42) | (1.40) |
| Age (group z) |  | 0.0130 | 0.00438 |  | -0.0176 | -0.0152 |
|  |  | (0.25) | (0.08) |  | (-0.32) | (-0.28) |
| HH Income (group z) |  | 0.234*** | 0.228*** |  | 0.0613 | 0.0571 |
|  |  | (4.47) | (4.37) |  | (1.12) | (1.04) |
| Trust in Institutions (group z) |  |  | 0.122** |  |  | -0.101* |
|  |  |  | (2.81) |  |  | (-2.40) |
| Central Bank # Trust in Central Bank (group z) |  |  | -0.100 |  |  | 0.0956 |
|  |  |  | (-1.82) |  |  | (1.81) |
| Tech Company # Trust in Tech Companies (group z) |  |  | -0.0256 |  |  | 0.0957 |
|  |  |  | (-0.46) |  |  | (1.79) |
| intercept | 4.552*** | 4.431*** | 4.482*** | 4.228*** | 4.150*** | 4.111*** |
|  | (57.60) | (46.32) | (46.50) | (54.08) | (42.88) | (42.00) |
|  |  |  |  |  |  |  |
| var(intercept) | 2.620^***^ | 2.551^***^ | 2.492^***^ | 2.893^***^ | 2.890^***^ | 2.889^***^ |
|  | (19.39) | (18.71) | (17.93) | (22.06) | (21.96) | (21.69) |
| var(Residual) | 1.528^***^ | 1.522^***^ | 1.512^***^ | 1.390^***^ | 1.387^***^ | 1.373^***^ |
|  | (14.52) | (14.31) | (13.82) | (11.26) | (11.16) | (10.58) |
| Observations | 3,515 | 3,488 | 3,398 | 3,515 | 3,488 | 3,398 |

*t* statistics in parentheses; ^*^ *p* < 0.05, ^**^ *p* < 0.01, ^***^ *p* < 0.001

**Table S5: Estimation Results for the Country-specific Analyses for USA**

|  | Digital Currency Usage | | | Propensity of Data Breach | | |
| --- | --- | --- | --- | --- | --- | --- |
|  | M13 | M14 | M15 | M16 | M17 | 18 |
| Central Bank | -0.0236 | -0.0236 | 0.00641 | 0.0456 | 0.0430 | 0.0115 |
|  | (-0.43) | (-0.43) | (0.11) | (1.13) | (1.06) | (0.26) |
| Tech Company | -0.407^***^ | -0.407^***^ | -0.386^***^ | 0.402^***^ | 0.403^***^ | 0.383^***^ |
|  | (-7.44) | (-7.44) | (-6.70) | (9.93) | (9.96) | (8.88) |
| Low Popularity | -0.261^***^ | -0.261^***^ | -0.279^***^ | 0.117^**^ | 0.113^**^ | 0.113^**^ |
|  | (-4.79) | (-4.77) | (-4.90) | (2.89) | (2.80) | (2.66) |
| High Popularity | 0.119^*^ | 0.120^*^ | 0.101 | -0.0508 | -0.0518 | -0.0552 |
|  | (2.18) | (2.19) | (1.78) | (-1.26) | (-1.28) | (-1.30) |
| VAT | 0.293^***^ | 0.294^***^ | 0.324^***^ | -0.0312 | -0.0272 | -0.0280 |
|  | (5.37) | (5.37) | (5.69) | (-0.77) | (-0.67) | (-0.66) |
| Pension Funds | 0.404^***^ | 0.404^***^ | 0.421^***^ | -0.106^**^ | -0.103^*^ | -0.106^*^ |
|  | (7.39) | (7.39) | (7.39) | (-2.63) | (-2.54) | (-2.48) |
| Provider Sees All | -0.132^*^ | -0.132^*^ | -0.121^*^ | 0.124^**^ | 0.124^**^ | 0.139^**^ |
|  | (-2.42) | (-2.41) | (-2.12) | (3.06) | (3.07) | (3.25) |
| End‑of‑Month Overview | -0.0137 | -0.0143 | -0.0180 | 0.0162 | 0.0129 | 0.0295 |
|  | (-0.25) | (-0.26) | (-0.32) | (0.40) | (0.32) | (0.69) |
| Male |  | 0.109 | 0.0939 |  | -0.0633 | -0.0717 |
|  |  | (1.10) | (0.93) |  | (-0.69) | (-0.78) |
| Age (group z) |  | -0.104^*^ | -0.0999 |  | 0.209^***^ | 0.193^***^ |
|  |  | (-2.06) | (-1.93) |  | (4.43) | (4.07) |
| HH Income (group z) |  | 0.120^*^ | 0.115^*^ |  | 0.131^**^ | 0.126^**^ |
|  |  | (2.36) | (2.20) |  | (2.77) | (2.62) |
| Trust in Institutions (group z) |  |  | 0.189^***^ |  |  | -0.172^***^ |
|  |  |  | (3.53) |  |  | (-4.04) |
| Central Bank # Trust in Central Bank (group z) |  |  | -0.181^**^ |  |  | 0.117^**^ |
|  |  |  | (-3.01) |  |  | (2.58) |
| Tech Company # Trust in Tech Companies (group z) |  |  | -0.101 |  |  | 0.0616 |
|  |  |  | (-1.72) |  |  | (1.40) |
| intercept | 3.571^***^ | 3.516^***^ | 3.493^***^ | 4.972^***^ | 5.004^***^ | 5.040^***^ |
|  | (44.55) | (37.68) | (36.20) | (75.34) | (63.11) | (62.17) |
|  |  |  |  |  |  |  |
| var(intercept) | 2.309^***^ | 2.279^***^ | 2.283^***^ | 2.258^***^ | 2.181^***^ | 2.084^***^ |
|  | (16.10) | (15.78) | (15.30) | (17.33) | (16.50) | (14.82) |
| var(Residual) | 1.780^***^ | 1.783^***^ | 1.774^***^ | 0.976 | 0.973 | 0.991 |
|  | (19.93) | (19.96) | (18.88) | (-0.84) | (-0.94) | (-0.31) |
| Observations | 3,581 | 3,575 | 3,344 | 3,580 | 3,574 | 3,343 |

*t* statistics in parentheses; ^*^ *p* < 0.05, ^**^ *p* < 0.01, ^***^ *p* < 0.001

**Table S6: Digital Currency Usage - Estimation Results for Multilevel Regression with Country Fixed Effects**

|  | M19 | M20 | M21 |
| --- | --- | --- | --- |
| Central Bank | -0.00576 | -0.00365 | 0.00670 |
|  | (-0.19) | (-0.12) | (0.21) |
| Tech Company | -0.426^***^ | -0.426^***^ | -0.444^***^ |
|  | (-13.69) | (-13.68) | (-13.61) |
| Low Popularity | -0.174^***^ | -0.179^***^ | -0.192^***^ |
|  | (-5.60) | (-5.74) | (-5.98) |
| High Popularity | 0.111^***^ | 0.110^***^ | 0.0901^**^ |
|  | (3.56) | (3.52) | (2.80) |
| VAT | 0.279^***^ | 0.276^***^ | 0.287^***^ |
|  | (8.97) | (8.87) | (8.92) |
| Pension Funds | 0.332^***^ | 0.332^***^ | 0.332^***^ |
|  | (10.68) | (10.68) | (10.30) |
| Provider Sees All | -0.153^***^ | -0.153^***^ | -0.146^***^ |
|  | (-4.91) | (-4.91) | (-4.55) |
| End‑of‑Month Overview | 0.00145 | 0.00327 | 0.00838 |
|  | (0.05) | (0.11) | (0.26) |
| India | 1.286^***^ | 1.543^***^ | 1.427^***^ |
|  | (17.70) | (18.55) | (16.40) |
| USA | 0.329^***^ | 0.422^***^ | 0.375^***^ |
|  | (4.55) | (5.30) | (4.61) |
| HH Income (grand z) |  | 0.446^***^ | 0.407^***^ |
|  |  | (4.28) | (3.86) |
| India # HH Income (grand z) |  | -0.203 | -0.172 |
|  |  | (-1.79) | (-1.51) |
| USA # HH-Income (grand-z) |  | -0.304^**^ | -0.271^*^ |
|  |  | (-2.60) | (-2.29) |
| Male |  | 0.224^***^ | 0.224^***^ |
|  |  | (3.82) | (3.80) |
| Age (grand z) |  | -0.196^***^ | -0.194^***^ |
|  |  | (-6.61) | (-6.47) |
| Trust in Institutions (grand z) |  |  | 0.169^***^ |
|  |  |  | (5.42) |
| Central Bank # Trust in Central Bank (grand z) |  |  | -0.116^***^ |
|  |  |  | (-3.38) |
| Tech Company # Trust in Tech Companies (grand z) |  |  | -0.0281 |
|  |  |  | (-0.84) |
| intercept | 3.247^***^ | 3.009^***^ | 3.080^***^ |
|  | (51.61) | (40.05) | (39.76) |
|  |  |  |  |
| var(intercept) | 2.516^***^ | 2.429^***^ | 2.376^***^ |
|  | (31.35) | (29.90) | (28.22) |
| var(Residual) | 1.707^***^ | 1.703^***^ | 1.710^***^ |
|  | (31.78) | (31.59) | (30.65) |
| Observations | 10,592 | 10,556 | 10,026 |

**Table S7: Propensity of Data Breach - Estimation Results for Multilevel Regression with Country Fixed Effects**

|  | M22 | M23 | M24 |
| --- | --- | --- | --- |
| Central Bank | 0.00870 | 0.00804 | -0.00710 |
|  | (0.33) | (0.31) | (-0.26) |
| Tech Company | 0.499^***^ | 0.503^***^ | 0.514^***^ |
|  | (18.98) | (19.14) | (18.57) |
| Low Popularity | 0.0477 | 0.0484 | 0.0488 |
|  | (1.81) | (1.84) | (1.79) |
| High Popularity | -0.0819^**^ | -0.0819^**^ | -0.0729^**^ |
|  | (-3.11) | (-3.12) | (-2.67) |
| VAT | -0.0265 | -0.0236 | -0.0193 |
|  | (-1.01) | (-0.90) | (-0.70) |
| Pension Funds | -0.0705^**^ | -0.0699^**^ | -0.0652^*^ |
|  | (-2.68) | (-2.66) | (-2.39) |
| Provider Sees All | 0.122^***^ | 0.124^***^ | 0.131^***^ |
|  | (4.64) | (4.70) | (4.80) |
| End‑of‑Month Overview | 0.0401 | 0.0353 | 0.0436 |
|  | (1.52) | (1.34) | (1.60) |
| India | -0.761^***^ | -0.808^***^ | -0.670^***^ |
|  | (-11.03) | (-10.14) | (-8.14) |
| USA | 0.110 | -0.0227 | 0.0485 |
|  | (1.61) | (-0.30) | (0.63) |
| HH Income (grand z) |  | -0.278^**^ | -0.243^*^ |
|  |  | (-2.79) | (-2.43) |
| India # HH Income (grand z) |  | 0.310^**^ | 0.272^*^ |
|  |  | (2.86) | (2.51) |
| USA # HH Income (grand z) |  | 0.437^***^ | 0.396^***^ |
|  |  | (3.91) | (3.52) |
| Male |  | -0.0606 | -0.0699 |
|  |  | (-1.08) | (-1.25) |
| Age (grand z) |  | 0.121^***^ | 0.112^***^ |
|  |  | (4.27) | (3.91) |
| Trust in Institutions (grand z) |  |  | -0.190^***^ |
|  |  |  | (-7.03) |
| Central Bank # Trust in Central Bank (grand z) |  |  | 0.122^***^ |
|  |  |  | (4.16) |
| Tech Company # Trust in Tech Companies (grand z) |  |  | 0.0363 |
|  |  |  | (1.27) |
| intercept | 4.854^***^ | 4.964^***^ | 4.896^***^ |
|  | (84.20) | (70.88) | (68.30) |
|  |  |  |  |
| var(intercept) | 2.373^***^ | 2.341^***^ | 2.259^***^ |
|  | (30.84) | (30.26) | (27.94) |
| var(Residual) | 1.221^***^ | 1.214^***^ | 1.229^***^ |
|  | (11.84) | (11.52) | (11.77) |
| Observations | 10,592 | 10,556 | 10,026 |

**SI References**

1. OECD. *Purchasing power parities (PPP) (indicator)*. (Accessed on 27 October 2022) (2022) doi:doi: 10.1787/1290ee5a-en.

2. OECD. What are equivalence scales? *OECD Proj. Income Distrib. Poverty* (2011).
